# A viral movement protein mediates host volatile biosynthesis to co-attract vectors and non-vectors and enhances viral infection

**DOI:** 10.3389/fpls.2025.1551362

**Published:** 2025-05-16

**Authors:** Shijun Li, Xiangwen Luo, Yu Zhang, Zhanhong Zhang, Xiao Yang, Xuguo Zhou, Xin Wang, Xian OuYang, Xiaoxin Qu, Deyong Zhang, Songbai Zhang, Yong Liu

**Affiliations:** ^1^ College of Plant Protection, Hunan Agricultural University, Changsha, China; ^2^ Key Laboratory of Pest Management of Horticultural Crop of Hunan Province, Institute of Plant Protection of Hunan Academy of Agricultural Science, Changsha, China; ^3^ Yuelushan Laboratory, Changsha, China; ^4^ Department of Entomology, School of Integrative Biology, College of Liberal Arts and Sciences, University of Illinois Urbana-Champaign, Urbana, IL, United States; ^5^ Longping Branch, Biology College of Hunan University, Changsha, China; ^6^ Institute of Bast Fiber Crops of Chinese Academy of Agricultural Sciences, Changsha, China

**Keywords:** pepper veinal yellows virus, vector, non-vector, volatiles, tetra-interaction

## Abstract

The literature shows that vector-transmitted plant viruses mediate host volatile biosynthesis to attract vectors and repel non-vectors, benefiting plant-infecting viruses and enhancing their spread. In this study, pepper veinal yellows virus (PeVYV) and its encoded movement protein P4 were shown to mediate host volatile biosynthesis to co-attract its exclusive vector aphids and non-vector Q-whiteflies; both have been proven to have existing food competition. The P4 protein enhances the biosynthesis of three volatiles and reduces the biosynthesis of one volatile involved in co-attracting and co-repelling aphids and Q-whiteflies. Viral replication is enhanced significantly in plants co-fed on by aphids and Q-whiteflies compared with plants fed on individual insects. Viral replication is enhanced significantly in the early stage for plants fed on by Q-whiteflies while in the late stage for plants fed on by aphids. Trans-zeatin (tZ) biosynthesis was increased in plants fed on by aphids and Q-whiteflies, and tZ enhanced viral replication in these plants. These data suggest that the PeVYV P4 protein mediates host volatile biosynthesis to co-attract vectors and non-vectors, benefiting viral infection probably by enhancing tZ biosynthesis. Data in this study have broad implications regarding the ecological significance of whiteflies in various agricultural ecosystems where aphids are the key viral transmitters.

## 1 Introduction

Plants constantly experience abiotic and biotic threats that seriously damage the sustainability of yields in cultivated fields. Among biotic threats, plant viruses and their vectors that attack plants are common in nature (Scholthof et al., 2011). Hemipterans, such as aphids, whiteflies, and leafhoppers, are devastating insect pests because they directly feed on plants and transmit numerous plant pathogens, including plant viruses. For example, aphids feeding on peppers transmit pepper veinal yellows virus (PeVYV), whereas whiteflies feeding on peppers transmit the pepper yellow leaf curl Indonesia virus (PepYLCIV) and a polerovirus pepper whitefly-borne vein yellows virus (Ghosh et al., 2019; Nasruddin et al., 2020; Cunniffe et al., 2021). In addition, vectors are not merely passive syringes injecting viruses into plants; they also promote virus epidemiology (Shi et al., 2021), and reciprocally, transmitted viruses may regulate a plant’s immune system to benefit the vector by interacting with plants (Li et al., 2019). Some transmitted viruses replicate in vectors and regulate their immune system and behavior to enhance the spread of the virus, which requires interaction with the vector (He et al., 2020; Zhang et al., 2021). Throughout evolutionary history, virus–plant–vector systems have developed complicated relationships with important ecological and evolutionary implications.

The complicated relationships of virus–plant–vector systems have been studied extensively. A virus-infected host may promote the performance of the associated insect vectors to facilitate their own transmission and spread (Li et al., 2014; Wu et al., 2019); however, the possible effects of these viruses on non-vector insects have received a paucity of consideration. Cucumber mosaic virus (CMV) is transmitted via aphids and mechanical routes. CMV-infected chili plants (*Capsicum annum* L.) attract the CMV vector aphid (*Myzus persicae*) and repel the non-vector sweet potato whitefly [*Bemisia tabaci* (Gennadius) (Hemiptera: Aleyrodidae)], and reduce its adaptation, resulting in whiteflies tending toward healthy chili plants (Saad et al., 2019). Tomato spotted wilt virus is transmitted by thrips (*Frankliniella occidentalis*), and infecting chili plants enhances the survival and oviposition of non-vector two-spotted spider mite *Tetranychus urticae* but reduces the fecundity and longevity of *B. tabaci* biotype Q (Q-whitefly) (Belliure et al., 2010; Pan et al., 2013). The DNA virus cotton leaf curl Multan virus (CLCuMuV), which is exclusively transmitted by whitefly, reprograms plant immunity to promote the fitness of the vector and suppress the performance of non-vector insects, including cotton bollworms (*Helicoverpa armigera*) and aphids (Zhao et al., 2019). The complexity of tetra-interactions among virus–vector and non-vector–host occurs widely in various natural and agricultural ecosystems; however, minimal attention has been paid to how a virus deters host–vector and non-vector interactions to favor its infection of host plants.

Insects feeding or viral infection of plants activates the defense response of plants, and phytohormones are key initiators that function as central cellular signaling molecules that regulate plant immunity against insects and/or viruses (Pan et al., 2021; Wu and Ye, 2020). In addition, phytohormones coordinate plant growth, development, and physiology (Pieterse et al., 2012; Smith et al., 2017). These above findings demonstrate that phytohormones have pivotal roles in virus–vector–host interactions. Recent studies have shown that phytohormones also play a dominant role in virus–vector and non-vector–host interactions. For instance, the CLCuMuV βC1 protein interacts with transcription factor WRKY20 to mediate salicylic acid (SA) biosynthesis, benefiting viral replication in plants and vector whiteflies; however, it negatively affects non-vector competitors, including cotton bollworms (*H. armigera*) and aphids (Zhao et al., 2019). The phenomena of virus–vector and non-vector insects simultaneously infecting plants are common in natural and agroecological systems (Mandadi and Scholthof, 2013; Stout et al., 2006; Wu and Baldwin, 2010); thus, intensive interactions between viruses, their vectors, non-vectors, and plants are inevitable. These relationships are important both for individual organisms and for the population dynamics of each species involved in an ecosystem (Stout et al., 2006; Carr et al., 2018; Donnelly and Gilligan, 2020; Tack et al., 2013); therefore, elucidating the nature of these interactions in detail and translating them into knowledge of ecology, evolution, and plant defense will provide further useful information for developing new strategies against plant pathogens and pests.

PeVYV is a phloem-restrictive virus belonging to the genus *Polerovirus* (family Solemoviridae, formerly Luteoviridae) (Filardo et al., 2024). PeVYV is mainly transmitted by two aphids, *Aphis gossypii* (melon and cotton aphid) and *M. persicae* (Ghosh et al., 2021). PeVYV encodes the small-molecule P4, a movement protein mediating the movement of the virus between host cells (Li et al., 2020); however, minimal work has been conducted on the movement proteins in arboviruses, such as *polerovirus*, regarding virus–vector and non-vector, and host interactions.

Here, the *polerovirus* PeVYV is reported to alter plant volatiles via its encoded P4 protein to co-attract the vector aphid and non-vector Q-whitefly. Viral infection was enhanced by non-vector Q-whiteflies in the early stage of infection and exclusively by vector aphids in the late stage. The biosynthesis of the phytohormone trans-zeatin (tZ) induced by vectors and non-vectors likely plays a pivotal role in fine-tuning viral infection. The results uncover a hitherto uncharacterized ecological relationship of virus–vector and non-vector–host tetrapartite interactions, and the non-vector likely plays a crucial role in the vector-transmitted virus’ primary infection in plants. These findings could help to optimize practical strategies for effectively managing whiteflies and aphid populations in the field and also contribute to developing novel virus and pest management strategies applicable to diverse and complex agroecosystems.

## 2 Materials and methods

### 2.1 Plant and insect colonies

*Nicotiana tabacum* L. cv. Samsun NN and *Nicotiana benthamiana* wild-type (WT) transgenic line were planted in a greenhouse at 26°C–28°C and a 14-h light/10-h dark illumination cycle. The collected samples were immediately frozen in liquid nitrogen and stored at −80°C for further experiments except for special descriptions.

Non-viruliferous *B. tabaci* MEAM1/Q-whiteflies were generated and maintained on tobacco plants, and non-viruliferous *M. persicae* (Sulzer) green peach aphids were maintained on pepper (*C. annuum* L. var Zhongjiao 5) plants (Shi et al., 2016) in an insectary at 25°C ± 1°C, 70% ± 10% relative humidity, and a 14-h/10-h (light/dark) cycle (Shi et al., 2018).

### 2.2 Viruses and inoculation

PeVYV and PeVYV accompanying RNA (PeVYV-aRNA) full genomic cDNA infectious clones were kindly gifted by Dr. Jiejun Peng at Ningbo University (Ningbo, China). The viruses were inoculated onto plants using agrobacteria infiltration. The ratio of PeVYV-GV3101 to PeVYV-aRNA-GV3101 was 1:2 (Liu et al., 2021b).

### 2.3 Generation of P4-overexpressing transgenic plants

The full gene sequence of P4 was inserted into the pD1301S vectors and transformed into *Agrobacterium tumefaciens* GV3101 cells. The T-DNA of each P4 protein was introduced into the tobacco (*N. tabacum* L. cv. Samsun NN) genome using *A. tumefaciens*-mediated transformation. The positive lines were screened using hygromycin resistance and specific polymerase chain reaction (PCR) with the primers in **Supplementary Table S3**.

### 2.4 Growth physiology of tobacco plants

The plant height, leaf length, leaf width, and stem diameter of the tobacco plants were measured with rulers and vernier calipers. The chlorophyll content and photosynthetic rate of the tobacco leaves were quantified using a portable photosynthesis analyzer L1-6800 (LI-COR Inc., Lincoln, NE, USA) in a leaf chamber (6 cm^2^) at 30°C, 50% relative humidity, 800 µmol·m^−2^·s^−1^ light intensity, and 400 µmol m^−2^ s^−1^ CO_2_ concentration.

### 2.5 Extraction and analysis of volatiles

Volatile compounds were collected using headspace solid phase microextraction (HS-SPME) (Zhang et al., 2013; Tang et al., 2018). An accurately weighed mass of 0.5 g of leaves from 30-day-old P4 overexpressing transgenic- and WT *N. tabacum* L. cv. Samsun NN plants was placed in 1.5-mL vials, and then 2 μL of ethyl caprate (2.0 mg/mL) dissolved in ethyl alcohol was added as an internal standard. A 50/30-μm divinylbenzene/carboxen/polydimethylsiloxane (DVB/CAR/PDM) extraction head was inserted into the vial headspace. Headspace extraction was performed for 60 min, and sample desorption was performed at 260°C for 5 min in the gas chromatography (GC) injector inlet. Compound separation and identification were performed using a GC-mass spectrometer (MS) (Agilent 7890B-5977B, Agilent J&W scientific, Folsom, CA, USA) equipped with an ADB-wax fused-silica capillary column (30 m × 0.25 mm × 0.25 μm, Agilent J&W scientific, Folsom, CA, USA). The carrier gas was high-purity helium (purity not less than 99.999%), the constant flow rate was 1.0 mL/min, the injection port temperature was 260°C, no split flow injection, and the solvent delay was 1.5 min. Temperature programming: 40°C for 3 min, 5°C/min to 220°C, and hold for 5 min. The temperature of the MS ion source was 230°C, and the four-stage rod was 150°C; the electron energy was 70 eV. The scanning mode was full scan mode (scan), and the quality scanning range was m/Z: 20–650.

Volatile compounds were qualified using the NIST (National Institute of Standards and Technology, Gaithersburg, MD, USA) database. A data matrix was derived. The three-dimensional matrix includes sample information, the peak name of each substance, retention time, retention index, mass-to-charge ratio, and signal intensity. In each sample, all peak signal intensities were segmented and normalized according to the internal standards with a relative standard deviation greater than 0.3 after screening. After these data were normalized, redundancy removal and peak merging were conducted to obtain a data matrix.

The internal standard method was adopted to characterize the relative concentration of the individual volatiles quantitatively using the equation below:


$$content\;of\;component=\;\left(\frac{A1}{A2}\right)\;\;\times \;\left(M1/M2\right)$$

where A1 and A2 indicate the peak areas of a detected compound and the internal standard, respectively, and M1 and M2 refer to the added volumes of the internal standard and sample, respectively.

Principle component analysis was used to observe the overall distribution among the samples and the stability of the analysis process. Orthogonal partial least-squares-discriminant analysis (OPLS-DA) and partial least-squares-discriminant analysis (PLS-DA) were utilized to distinguish metabolites that differ between groups. To prevent overfitting, 7-fold cross-validation and 200 response permutation testing were used to evaluate the quality of the model. The variable importance of projection (VIP) values obtained from the OPLS-DA model were used to rank the overall contribution of each variable to group discrimination. A two-tailed Student’s *t*-test was used to verify further whether the metabolites differing between groups were significant. Differential metabolites were selected with VIP values greater than 1.0 and *ρ*-values less than 0.05.

### 2.6 Y-tube olfactometer choice assay

The olfactory response of whiteflies or aphids to host and volatiles (**Supplementary Table S2**) for test cues was tested using a glass Y-tube olfactometer purchased from Shanghai Hefan Instrument Co., Ltd. The Y-tube olfactometer described in Saad et al. (2015) and Morgan and Hare (2003) was used with minor modifications. Briefly, two streams of purified air, filtered through activated carbon, were passed through two glass containers into the olfactory measurement arm at a flow rate of 40 mL/min. The Y-tube olfactometer consisted of a 2.5-cm-diameter, 15-cm-long central tube and two 2.5-cm-diameter, 11-cm-long side arms with ground glass fitting. Humidified air was controlled via an upstream flow meter to pass through this ground glass fitting at 0.5 mL/s per sidearm. Each arm was connected to an extended glass tube 2 cm in diameter and 6 cm in length. An extended glass tube with a mesh barrier prevented the insects from escaping. The air was filtered through an activated carbon trap and then passed into a glass jar 18 cm in diameter and 30 cm in height. Each host plant leaf or volatile can be placed in the glass jar. The whole olfactometer system was pre-stabilized for a minimum of 2 h before use. The olfactometer was set at 25°C ± 1°C and 45%–55% relative humidity.

Volatile compounds were dissolved in n-hexane at final concentrations of 100 µL/mL or 100 mg/mL. For each test, 5 µL of the solution was applied to a filter paper (1.5 cm × 1.5 cm Whatman No. 1) that was sealed in an olfactometer. Insects were exposed to host or volatile treatment pairs (P4-OE plant leaves vs. WT plant leaves; volatile vs. n-hexane). Individuals were released within the first centimeter of the olfactometer tube arm, and their responses were measured for 1 h. Insects that climbed greater than 2/3 of the tube arm and did not return after 15 s were recorded as making a selection. Insects that did not choose within the 10-min limit were excluded from the results. Each experiment was repeated five times per plant or synthetic volatile organic compound (VOC), and each replicate consisted of 20 adult insects assayed individually (i.e., total n = 100 per treatment). To remove possible contamination of plant odors or volatiles from previous tests, the Y-tube was washed with 75% ethanol and cleaned using ddH_2_O thoroughly at least three times between tests.

### 2.7 RNA-seq and data analysis

Total RNA was extracted from 30-day-old tobacco leaves using the mirVana miRNA Isolation Kit (Ambion) following the manufacturer’s protocol. RNA integrity was evaluated using an Agilent 2100 Bioanalyzer (Agilent Technologies, Santa Clara, CA, USA). Six libraries were constructed using TruSeq Stranded mRNA LTSample Prep Kit (Illumina, San Diego, CA, USA) according to the manufacturer’s instructions and sequenced by OE Biotech Company (Shanghai, China) on an Illumina sequencing platform (HiSeq 2500 or Illumina HiSeq X Ten) and 125-bp/150-bp paired-end reads were generated. The clean reads were screened and mapped to the reference genome using HISAT2 (Kim et al., 2015), and the differentially expressed genes (DEGs) were identified using DESeq (Frazee et al., 2014). Gene ontology enrichment and Kyoto Encylopedia of Genes and Genomes (KEGG) pathway enrichment analyses of DEGs were performed using R based on the hypergeometric distribution (Kanehisa et al., 2008).

### 2.8 Total RNA isolation and quantitative reverse transcription polymerase chain reaction

Total RNA was isolated from tobacco leaf samples (100 mg tissue per sample) using TRIzol Up Reagent (TransGEN Biotech, Beijing, China). The concentration of total RNA in each sample was determined using a NanoDrop 2000 Spectrophotometer (Thermo Fisher Scientific, Wilmington, DE, USA). cDNA was synthesized using 1 µg total RNA per 20 µL reaction using the MonScript™ RTIII Super Mix with dsDNase (Two-Step) (Monad, Suzhou, China). Quantitative RT-PCRs were performed on an ABI PRISM 7500 device using a TransStart Green qPCR SuperMix UDG Kit (TransGEN). The relative transcript levels were calculated using the 2^−ΔΔ^Ct method as previously described, and the *N. benthamiana actin* and *GAPDH* genes were used as internal controls. The primers for quantitative RT-PCR analysis are listed in **Supplementary Table S3**.

### 2.9 Quantification of endogenous phytohormones in tissue samples

A total of healthy adults comprising 10 aphids, 10 Q-whiteflies, or 10 aphids + 10 Q-whiteflies (male/female ratio 1:1) were placed on each of four true leaves of tobacco seedlings for continuous feeding. Five individual tobacco seedlings were set per treatment, and each treatment was replicated three times (i.e., n = 15). Tobacco plant 4th leaves were sampled, 50 mg per plant, at 10 and 20 days after insect inoculation, frozen with liquid nitrogen, and stored at −80°C. The samples were ground individually in liquid nitrogen and homogenized in 1-mL extraction buffer containing isopropanol/H_2_O/hydrochloric acid (200:100:0.2) and then incubated at −20°C for 12 h. Afterward, the samples were ultrasonicated for 30 min in an ice bath, followed by the addition of 1 mL of dichloromethane and 1 mL of 300 ng/mL double internal standard samples (succinic acid-2,2,3,3-d4 and Lyso PC17:0). The organic phase was evaporated to dryness in vacuo, dissolved in 200 mL methanol/H_2_O (5:95, including 10 ng/mL 2-cl-phe), ultrasonicated for 3 min in an ice bath, and centrifuged at 13,000 rpm for 10 min at 4°C. The supernatant of each sample was filtered through a 0.22-µm organic filter membrane. A volume of 150 μL of supernatant was taken by a brown LC injection vial and analyzed using the UPLC-ESI-MS/MS at OE Biotech (Shanghai, China) to detect the target metabolites qualitatively and quantitatively as described previously (Lu et al., 2023; Novak and Flokova, 2018).

### 2.10 Exogenous tZ and pharmaceutical inhibitor spraying

The phytohormone tZ was dissolved in ddH_2_O to prepare a solution of 20 ppm. Lovastatin was dissolved in a small volume of methanol, titrated to 50 mg/mL in ddH_2_O, stored at −20°C, diluted 1000-fold when used, and added with one drop of Tween-20 (Lan et al., 2016). The solution of tZ or its pharmaceutical inhibitors was sprayed on the leaf of a tobacco plant inoculated with PeVYV, and ddH_2_O was used as a control. The third leave of each tobacco plant was sampled at 10, 15, 20, and 25 days. Each treatment consisted of 5 individual *N. tabacum* plants, i.e., n = 5. PeVYV genomic RNA accumulation was quantified using RT-qPCR.

### 2.11 Statistical analysis

All data are presented as the mean ± standard deviation. Statistical analyses were performed with Data Processing System software (Tang and Zhang, 2012), IBM SPSS software, and GraphPad Prism software. Statistical analysis was performed using Student’s *t-*test. *p< 0.05, **p< 0.01, ***p< 0.001, and ****p< 0.0001.

## 3 Results

### 3.1 P4 protein and PeVYV co-attract vectors and non-vectors

Previous findings proved that the P4 protein of PeVYV is a movement protein (Li et al., 2020). To characterize the P4 protein in virus–host interactions, transgenic *N. tabacum* constitutively expressing the *P4* gene under a 35S promoter (P4-OE) was generated, and two lines of the T2 generation (P4–1 and P4-3) showed that the *P4* gene was recombinant in the *N. tabacum* genome and expressed in P4-OE plants (**Supplementary Figures S1A, B**). The morphology, as well as the chlorophyll content and the photosynthetic rate of P4-OE lines, was similar to WT plants (**Supplementary Figures S1C–I**). These data suggested that no adverse effects occurred in response to constitutively overexpressing the *P4* gene in *N. tabacum*.

**Figure 1:**
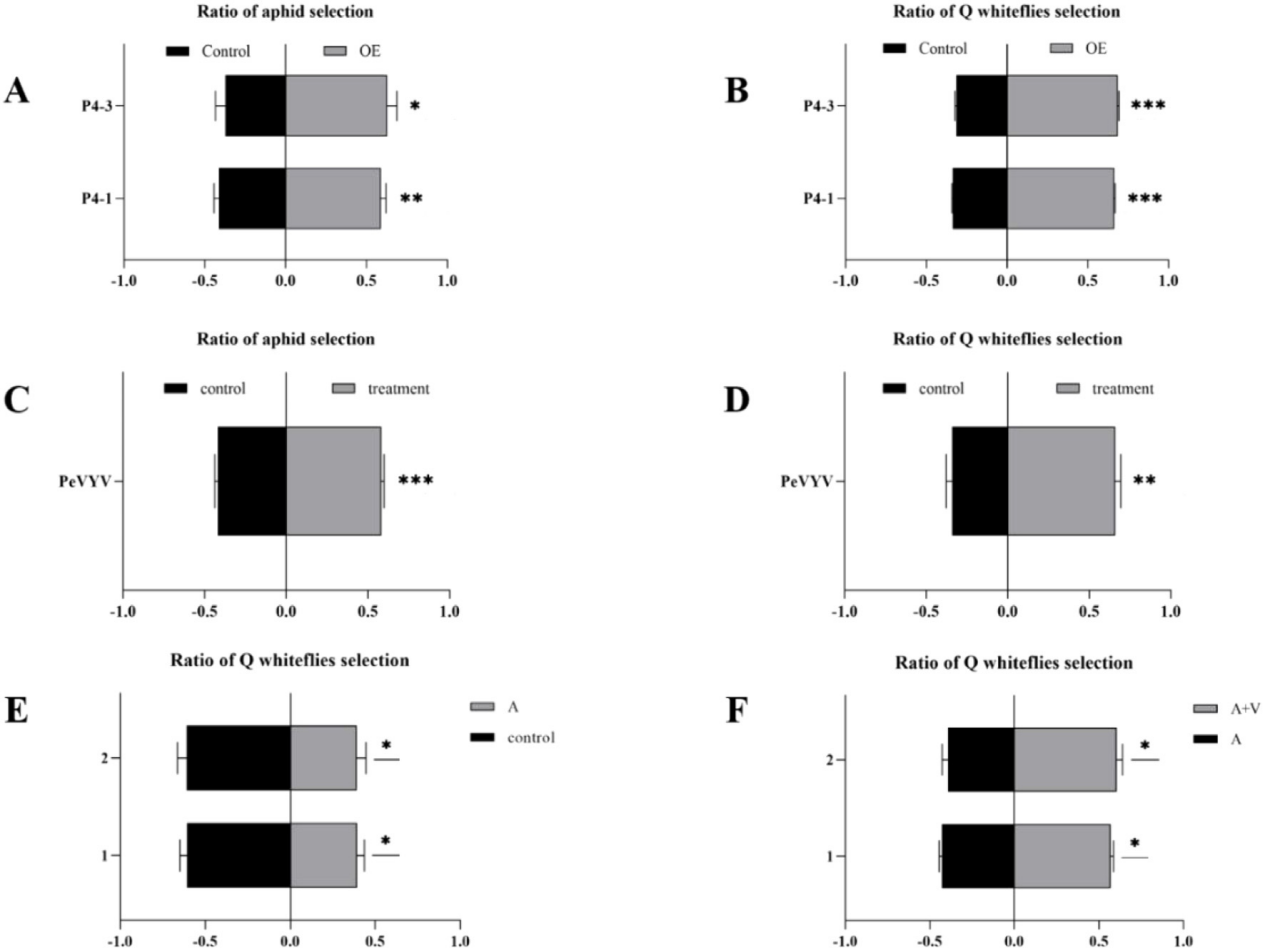
Pepper veinal yellows virus (PeVYV) and its encoded-P4 protein co-attract vector aphid and non-vector whitefly. **(A)** P4-OE *N. tabacum* plants attract aphids. **(B)** P4-OE *N tabacum* plants attract Q-whiteflies. **(C)** PeVYV-infected *N. tabacum* plants attract aphids. **(D)** PeVYV-infected *N. tabacum* plants attract Q-whiteflies. **(E)** Aphid-free peppers attract Q-whiteflies. **(F)** Peppers fed by virus-carrying aphids attract Q-whiteflies. **(A–F)** Bars represent means ± SD (each treatment replication for five times, and each replicate consisted of 20 adult insects assayed individually, i.e., total n = 100 for each treatment) (*p< 0.05, **p< 0.01, and ***p< 0.005, Student’s *t* test).

Unexpectedly, both Q-whiteflies and aphids opted for P4-OE plants over WT plants. Both Q-whiteflies and aphids are sap-sucking and have been recorded to have existing relationships of food competition (Ohgushi, 2012; Yamauchi et al., 2021). PeVYV was exclusively transmitted by aphids; the P4-OE plants overexpressing the *P4* gene encoded by PeVYV attracted aphids, likely benefiting viral transmission; however, the attraction of the vector’s competitor by plants infected with this vector-transmitted plant virus was not observed. Y-tube olfactometer choice assays were manipulated to confirm this phenomenon accurately. The two lines (P4–1 and P4-3) of P4-OE *N. tabacum* plants significantly attract virus–vector aphids (**Figure 1A**) and also significantly attract virus–non-vector Q-whiteflies (**Figure 1B**). To evaluate the possibility of this phenomenon existing in virus-infected plants, whether PeVYV-infected plants could attract aphids or Q-whiteflies was tested using Y-tube olfactometer choice assays. In addition to P4-OE *N. tabacum* plants, PeVYV-infected *N. tabacum* plants also significantly attracted aphids and Q-whiteflies (**Figures 1C, D**). To further estimate the possibility of this phenomenon existing in the field, whether Q-whiteflies tend toward non-viruliferous aphids or viruliferous aphids feeding plants was tested. The viruliferous aphids with PeVYV could effectively transmit the virus to peppers (**Supplementary Figures S2A, B**). Similarly to previous studies (Ohgushi, 2012; Yamauchi et al., 2021), non-viruliferous aphid-feeding plants repel Q-whiteflies (**Figure 1E**); however, viruliferous aphid-feeding plants that were infected with the transmitted virus PeVYV attracted Q-whiteflies (**Figure 1F**). In aggregate, these data verified that the P4 protein-overexpressing and PeVYV-infected plants co-attract the vector and non-vector.

### 3.2 Aphids and whiteflies enhance viral replication in plants

**Figure 2:**
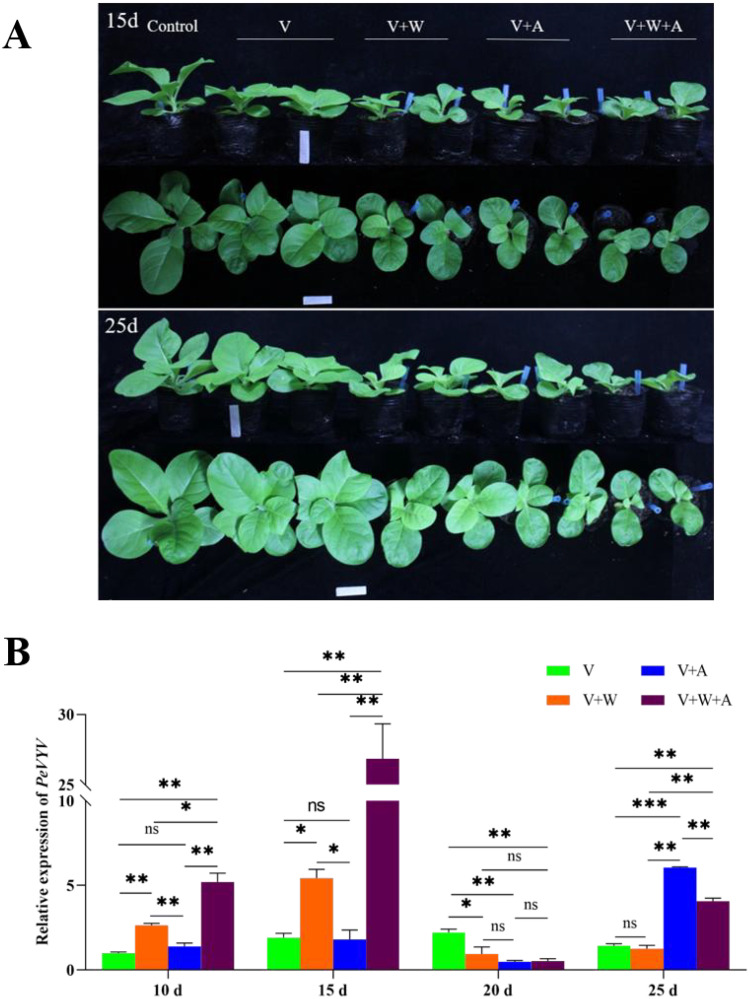
Aphids and/or whiteflies fine-tune PeVYV infection in plants. **(A)** Morphology of PeVYV infection plants affected by aphids and/or Q-whiteflies. **(B)** Virus genome accumulation of PeVYV infection plants affected by aphids and/or Q-whiteflies. Bars represent means ± SD (n = 3) (*p< 0.05, **p< 0.01, and ***p< 0.005, Student’s *t* test).

Vectors benefit transmitted viruses by aiding in infecting plants and vice versa (Belliure et al., 2005) and repelling non-vectors (Zhao et al., 2019). Why does PeVYV hijack the host to co-attract its vector aphid and non-vector whitefly, and which benefits viral infection? To answer this question, a PeVYV full genomic cDNA infectious clone was used to infiltrate the leaves of plants being fed on by aphids and/or Q-whiteflies, and the viral genome levels were quantified using RT-qPCR (**Figure 2**). Compared with healthy plants, slight dwarfism is displayed by PeVYV-infected plants; however, in PeVYV-infected plants fed on by Q-whiteflies and aphids/Q-whiteflies, significant dwarfism is displayed, whereas no dwarfism is observed in plants fed on by only aphids (**Figure 2A**; **Supplementary Figure S3A–C**). This demonstrates that Q-whiteflies, but not aphids, enhance PeVYV infection in plants in the early stage. Notably, more dwarfism occurred in PeVYV-infected plants fed on by aphids/Q-whiteflies compared with those fed on by only aphids or Q-whiteflies (**Figure 2A**; **Supplementary Figure S3A**), suggesting that the virus co-attracts the vector and the non-vector to enhance infection. Similarly, compared with healthy plants, slight dwarfism occurs in PeVYV-infected plants, suggesting that PeVYV infection without the vector has a slight effect on the host, whereas the severe dwarfism observed in PeVYV-infected plants fed on by aphids, Q-whiteflies, and aphids/Q-whiteflies (**Figure 2A**; **Supplementary Figures S3C–F**) demonstrates that aphids and Q-whiteflies enhance PeVYV infection in plants.

In addition, the genomic RNA accumulation of PeVYV in plants proved the symptoms of PeVYV-infected plants. The genomic RNA accumulation of PeVYV was slightly lower than in plants fed on by vector aphids; however, it was significantly higher in plants fed on by non-vector Q-whiteflies and was highest in plants fed on by both aphids/Q-whiteflies in the early stage of infection (from 10 to 15 dpi) compared with those not being fed on by insects (**Figure 2B**). These results demonstrate that non-vector Q-whiteflies enhance viral replication during early infection, which is critical for successful primary infection in plants. The contrary trend in the genomic RNA accumulation of PeVYV exists at 20 dpi, likely resulting from the defense responses of plants to rapid viral replication (Pumplin and Voinnet, 2013). Nevertheless, the genomic RNA accumulation of PeVYV was rapidly recovered in plants fed on by aphids and aphids/Q-whiteflies at 25 dpi; however, the genomic RNA accumulation in PeVYV-infected plants fed on by aphids/Q-whiteflies was lower than in plants fed on by aphids (**Figure 2B**), suggesting that vector aphids but not Q-whiteflies enhance viral infection in plants in the late stage. In aggregate, these data suggest that aphids and Q-whiteflies fine-tune PeVYV infection in plants.

### 3.3 The P4 protein reprograms the plant’s volatile biosynthesis pathways

**Figure 3:**
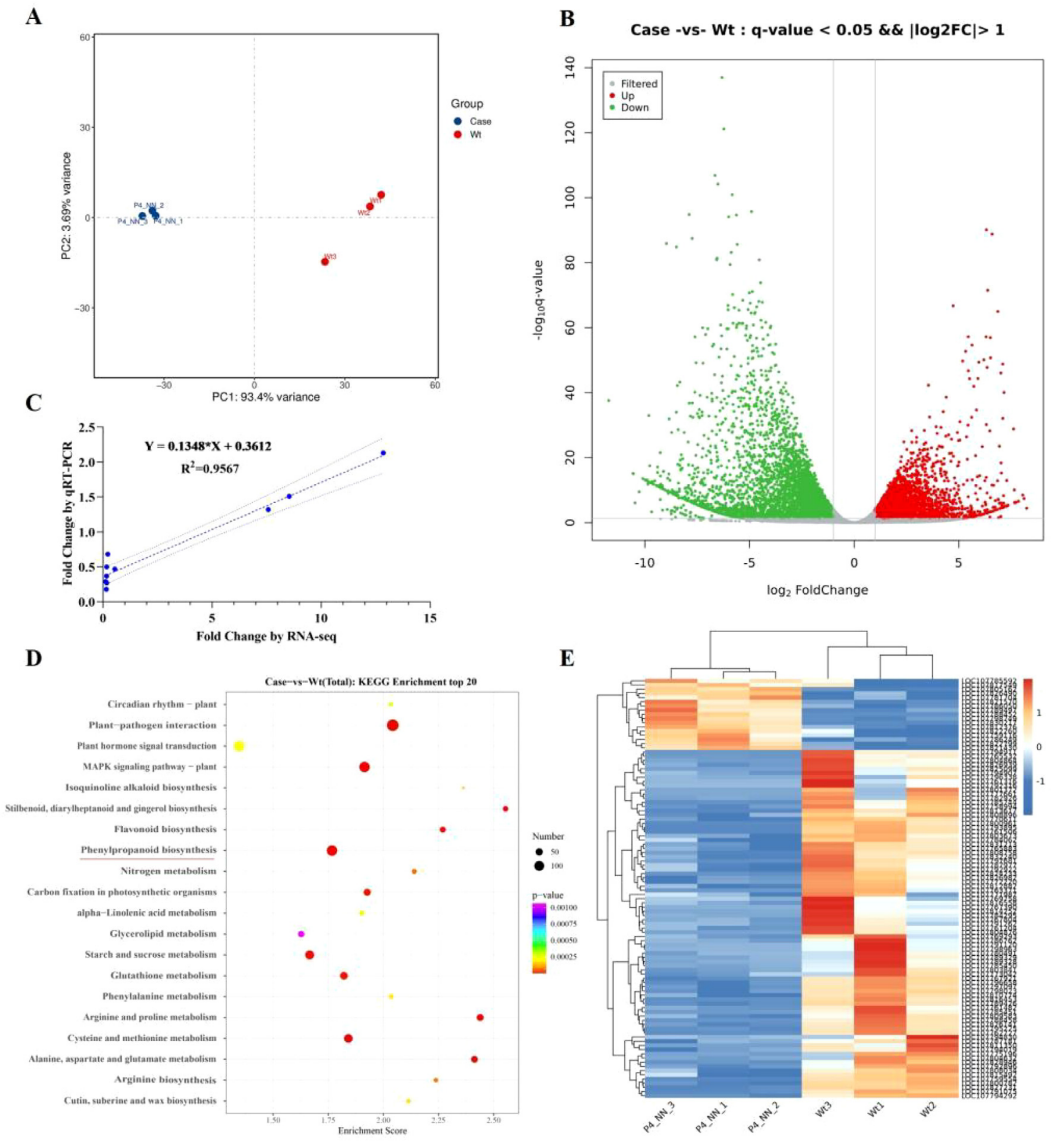
P4 protein reprograms the volatile biosynthesis pathway in *Nicotiana tabacum*. **(A)** Principal component analysis of leaf transcriptome data of between the P4-OE and the wild type of *N tabacum*. **(B)** The volcano map presents the deferentially expressed genes (FDR < 0.05 and ≥2-fold change) between the P4-OE and the wild type based on leaf transcriptome analysis. **(C)** Comparison of the fold changes of 10 selected transcripts using RNA-Seq and RT-qPCR. Each blue point represents a chosen gene used in the validation of the RNA-Seq results. **(D)** KEGG pathways with enrichment of significantly upregulated and downregulated genes. The phenylpropanoid biosynthesis pathway is underlined in red. **(E)** The heatmap presents the differentially expressed genes (FDR < 0.05 and ≥2-fold change) involved in the phenylpropanoid biosynthesis pathway.

The VOCs released by plants can function as chemical cues for many insect and animal interactions with plants (Allmann and Baldwin, 2010; Pichersky et al., 2006). Capitalizing on plant VOCs, vector-transmitting viruses regulate the pathways associated with plant VOC biosynthesis to release specialized VOCs and attract their vector (Wu et al., 2019). To elucidate the possible mechanisms by which the P4 protein hijacks host plant biosynthesis to attract vectors/non-vectors, RNA-seq analysis was performed to compare the transcription profiles of P4-OE *N. tabacum* plants with WT plants. The leaves of P4-OE (P4–1 line) and WT *N. tabacum* plants were sampled and sequenced. Compared with WT plants, a total of 8,157 DEGs were induced in the P4-OE plants, with 3,277 being upregulated and 4,880 downregulated (**Figure 3A**). The repeatability of the expression fold changes obtained in the RNA-seq dataset was validated with a high correlation coefficient (*R^2^* = 0.96) when comparing with selected genes (*LBD12, ATL79, GSTT3, MYB5, AOX1B, RGA5, NCED4, CHX15, CKX1*, and *WRKY27*) by RT-qPCR (**Figure 3B**). The KEGG annotation of DEGs revealed the enrichment of pathways related to virus–host interactions, including plant hormone signal transduction and the MAPK signaling pathway (**Figure 3C**). Notably, the phenylpropanoid biosynthesis pathway (nta00940, **Supplementary Figure S4**) was also induced in P4-OE plants (**Figure 3C**) and is strongly associated with plant metabolite synthesis, including VOC synthesis (Vogt, 2010). The expression profile of DEGs involved in the phenylpropanoid biosynthesis pathway (Madden et al., 2000; Mauck, 2016) shows that the majority of DEGs are unregulated (**Figure 3D, E**), suggesting that the P4 protein is probably involved in regulating plant VOC biosynthesis.

### 3.4 The P4 protein induces the plant’s volatile biosynthesis to co-attract aphids and whiteflies

**Figure 4:**
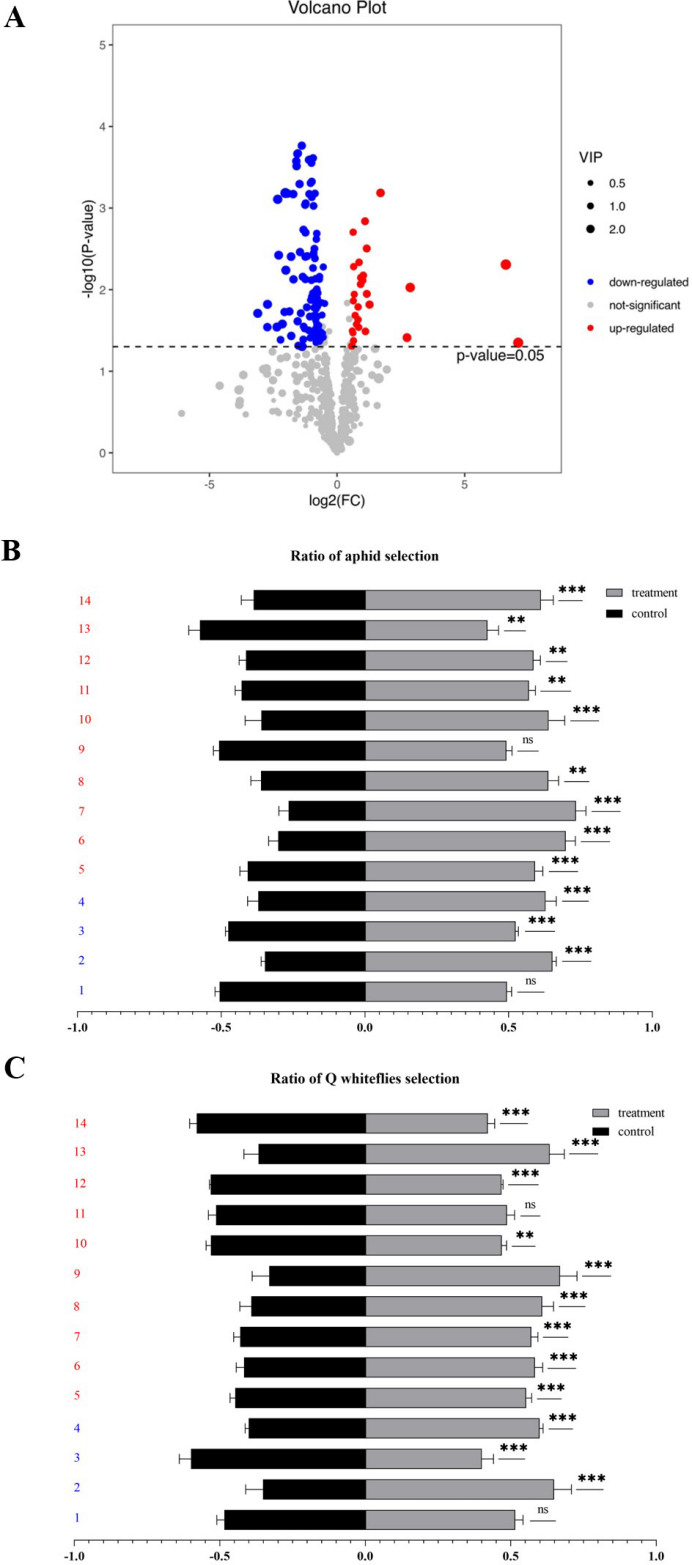
P4 protein hijacks the plant volatiles to co-attract aphids and Q-whiteflies. **(A)** The volcano map presents the differential content of volatiles (FDR< 0.05 and ≥2-fold change) between the P4-OE and the wild type based on volatile analysis. **(B)** Volatiles attract aphids by Y-tube tests. **(C)** Volatiles attract whiteflies by Y-tube tests. **(B, C)**. 1: Phytol, acetate; 2: boldenone benzoate; 3: topotecan; 4: 2,4-nonadienal, (e,e); 5: 3-cyclohexene-1-carboxaldehyde; 6: 1-cyclohexene-1-carboxaldehyde, 2,6,6-trimethyl; 7: sulfurous acid, dimethyl ester; 8: 4-ethyl-2-hydroxycyclopent-2-en-1-one; 9: 2-nonenal, (e)-; 10: 1-penten-3-ol; 11: 3-buten-2-one, 4-(2,6,6-trimethyl-1-cyclohexen-1-yl); 12: orcinol; 13: hexadecanoic acid, methyl ester; 14: 2(4h)-benzofuranone, 5,6,7,7a-tetrahydro-4,4,7a-trimethyl-, (r). Bars represent means ± SD (1–4 marked as blue represents down-releasing volatiles, 5–14 marked as red represents up-releasing volatiles. Each volatile test replication five times, and each replicate consisted of 20 adult insects assayed individually, i.e., total n = 100 for each volatile) (*p< 0.05, **p< 0.01, and ***p< 0.005, Student’s *t* test).

To investigate whether plant VOC biosynthesis was induced by the P4 protein, the VOCs released from P4-OE (P4–1 line) and WT plants were sampled using HS-SPME and identified by GC-MS. A total of 724 VOCs were identified in the WT and P4-OE plants (**Supplementary Table S1**), and compared with the WT plants, a total of 129 VOCs were significantly different in terms of biosynthesis and release in P4-OE plants (**Figure 4A**; **Supplementary Table S2**). Of these VOCs, 14 commercially available versions, including 10 showing enhanced release and four with reduced release, were selected to test aphid and Q-whitefly behaviors in the Y-tube olfactometer choice assays (**Figures 4B, C**). From the 10 VOCs showing enhanced release, four VOCs including 4-ethyl-2-hydroxycyclopent-2-en-1-one; sulfurous acid, dimethyl ester; 1-cyclohexene-1-carboxaldehyde, 2,6,6-trimethyl; and 3-cyclohexene-1-carboxaldehyde co-attracted aphids and Q-whiteflies, and the VOC topotecan, showing reduced release, co-repelled aphids and Q-whiteflies (**Figures 4B, C**). These data suggest that the P4 protein is possibly mediating plant VOC biosynthesis and emission to co-attract aphids and Q-whiteflies.

### 3.5 Aphids and Q-whiteflies co-feeding on plants enhance phytohormone trans-zeatin biosynthesis

**Figure 5:**
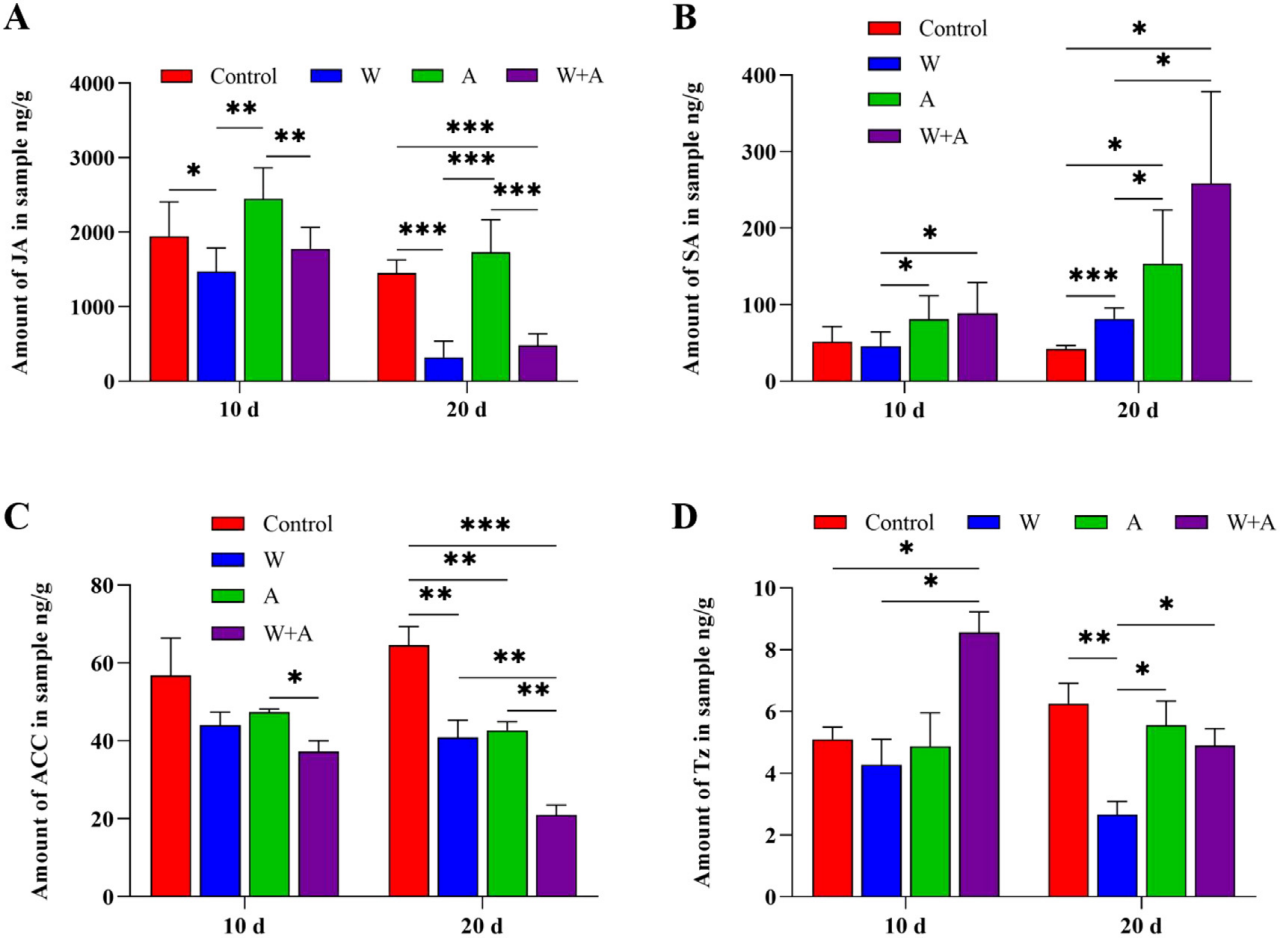
Plant hormone accumulation is affected by aphids and/or whiteflies in plants. **(A)** Jasmonic acid(JA). **(B)** 1-Aminocyclopropane-1-carboxylic acid (ACC). **(C)** Salicylic acid (SA). **(D)** Trans-zeatin (Tz). A total of 10 aphids, 10 Q whiteflies, or 10 aphids + 10 Q whiteflies of healthy adults (male/female ratio 1:1) were respectively placed on each of four true leaves of tobacco seedlings for continuous feeding. Five individual tobacco seedlings are set for each treatment, and each treatment is replicated for three times (i.e., n=15). The tobacco fourth leaves were sampled at 10 and 20 days after insect inoculation. Bars represent means ± SD (*p< 0.05, **p< 0.01, and ***p< 0.005, Student’s *t* test).

Insect feeding on as well as virus infection in plants often causes turbulence in hormone biosynthesis in plants, in turn affecting viral replication in plants (Zhao et al., 2019; Li et al., 2017). Thus, phytohormone biosynthesis was tested in plants fed on by aphids and/or Q-whiteflies. There was no effect on abscisic acid (ABA) biosynthesis in plants fed on by aphids and/or Q-whiteflies at 10 days and only significantly decreased in plants fed on by aphids compared with the control (plant not fed on by insects) at 20 days (**Supplementary Figure S5A**). Jasmonic acid (JA) and SA were unaffected in the early stage but induced in the late stage by aphid and/or whitefly feeding (**Figures 5A, B**). The compound 1-aminocyclopropane-1-carboxylic acid (ACC) was unaffected in the early stage but inhibited in the late stage by aphid and/or whitefly feeding (**Figure 5C**). tZ was significantly induced by joint aphid/Q-whitefly feeding in the early stage but relatively inhibited by aphids in the late stage (**Figure 5D**). Q-whitefly and aphid individual feeding significantly inhibited ACC biosynthesis compared with no insect feeding at 10 and 20 days (**Figure 5C**). Indoleacetic acid (IAA) biosynthesis was unaffected at 20 days by aphid and/or Q-whitefly feeding (**Supplementary Figure S5B**). JA biosynthesis was raised in plants fed on by aphids but decreased in plants fed on by Q-whiteflies and Q-whiteflies/aphids at 10 and 20 days compared with the control (**Figure 5A**). The SA content was increased by aphids or Q-whiteflies and increased more significantly by aphids/Q-whiteflies (**Figure 5B**). Aphid/Q-whitefly joint feeding significantly raised the tZ accumulation at 10 days, and Q-whitefly feeding inhibited tZ biosynthesis significantly at 20 days (**Figure 5D**). The expression of the key genes involved in these hormones’ biosynthesis pathways was quantified using RT-qPCR (**Supplementary Figure S6**). The expression levels of the key genes *NtLOX*, *NtAOS*, and *NtAOC* involved in JA biosynthesis (Wasternack and Song, 2017) were not relatively different in response to insect feeding compared with the control at 10 days and were relatively upregulated by aphid feeding at 20 d (**Supplementary Figures S6A–C**). The expression levels of the key genes *NtSGT-1* and *PAL*3 involved in SA biosynthesis (Reichert et al., 2009; Seto et al., 2011; Pokotylo et al., 2022) were relatively upregulated by aphid or whitefly feeding and aphid/whitefly co-feeding at 10 days (**Supplementary Figures S6D–F**). Compared with the control, the expression levels of the key genes *ACS1-det* and *Ein2-det*, involved in ACC biosynthesis (Wang et al., 2019), were relatively upregulated. Nevertheless, another key gene, *ACO1-det*, was relatively downregulated by aphid feeding or aphid and whitefly co-feeding (**Supplementary Figures S6G–I**). The expression levels of the key genes *CYP735A1*, *CYP735A2*, and *ABCG21* in the tZ biosynthesis pathway (Cai et al., 2018; Takei et al., 2004; Zhao et al., 2023; Zhang et al., 2014) were relatively upregulated by aphid, whitefly, and aphid/whitefly feeding at 10 and 20 days (**Supplementary Figures S6J–L**). These data suggest that aphids and Q-whiteflies mediate phytohormone tZ biosynthesis.

### 3.6 tZ enhances viral infection in plants

**Figure 6:**
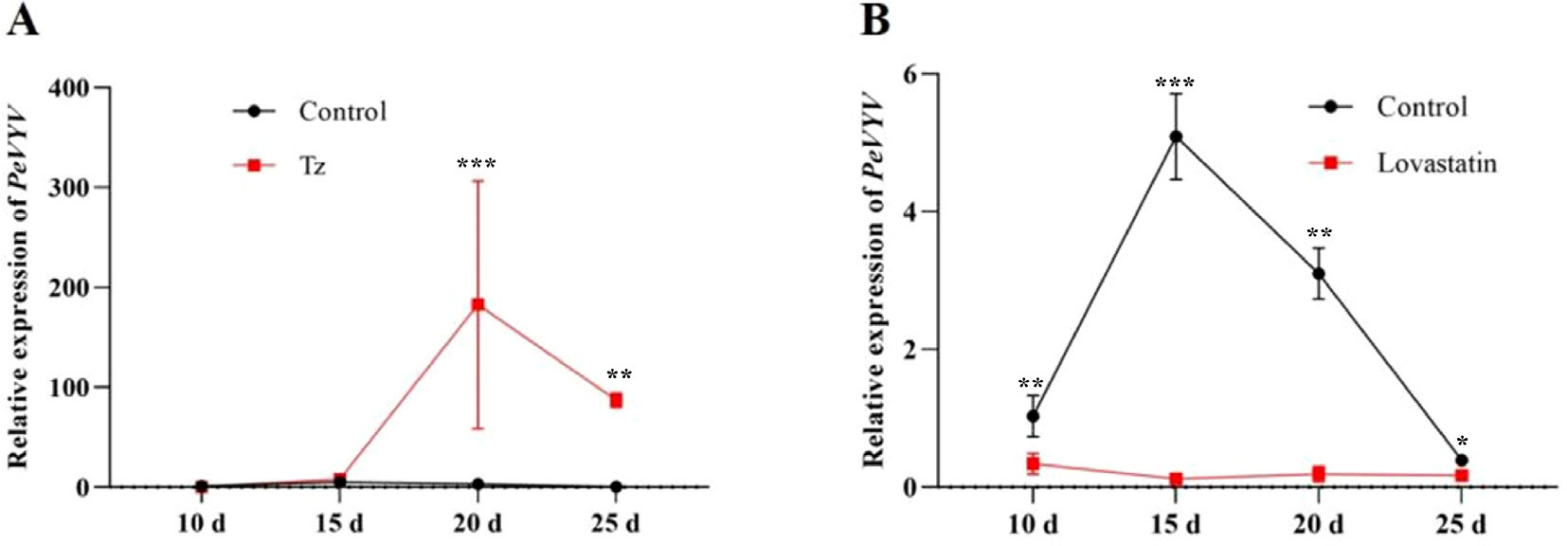
Plant hormones mediate PeVYV infection in plants. **(A)** The PeVYV genome RNA accumulation is affected by the exogenous spraying of Tz. **(B)** The PeVYV genome RNA accumulation is affected by spraying Tz biosynthesis inhibitors. **(A, B)** Each treatment consisted of five individual *N. tabacum* plants, i.e., n = 5, the third plant leaves were sampled at 10 to 20 days after phytohormones or pharmaceutical inhibitor spraying. Bars represent means ± SD (n = 5) (*p< 0.05, **p< 0.01, and ***p< 0.005, Student’s *t* test).

Based on the viral replication (**Figure 2B**) and phytohormone biosynthesis (**Figure 5**) affected by aphid/Q-whitefly feeding, it was speculated that tZ plays a pivotal role in viral replication. To test this hypothesis, viral replication affected by exogenous tZ spraying or its biosynthesis inhibitors was tested. The genomic RNA level of PeVYV was increased significantly in plants (from 20 to 25 days) by exogenous tZ spraying (**Figure 6A**). In contrast, the genomic RNA levels of PeVYV were decreased by exogenous spraying inhibitors of tZ biosynthesis (**Figure 6B**). These data suggest that tZ enhances viral infection in plants.

## 4 Discussion

The majority of plant viruses are transmitted by vectors, and vector–plant–virus interactions play a critical role in the spread and outbreak of viral diseases (Shi et al., 2021; He et al., 2020; Zhang et al., 2021). The complexity of vector–plant–virus interactions has been studied extensively and paved the way to understanding the epidemiology underlying vector-transmitted viruses (Cunniffe et al., 2021). In addition to the tri-interaction of virus–vector–host, the tetra-interaction of virus–vector and non-vector–plant could impact all involved parties; however, minimal attention has been paid to primary viral infections in the host. Recently, some attention has been paid to the effects of viral infection in plants by non-vector insects. These studies have drawn different conclusions but demonstrated that virus-infected plants had a great effect on non-vectors (Belliure et al., 2010; Pan et al., 2013; Zhao et al., 2019). As a result, it is rational to deduce that the non-vector probably impacts the virus; however, this has never been a concern until now. This study is a case in point presenting a virus exclusively transmitted by aphids; PeVYV reprogrammed the plant volatile biosynthesis pathway to deter non-vector whitefly behavior, combined with aphids to coordinate PeVYV infection in plants (**Figures 1**, **2**). Intriguingly, while aphids are common vectors for poleroviruses, PWVYV represents an exception as it is specifically transmitted by whiteflies (Ghosh et al., 2019). This implies potential host-vector co-evolutionary pathways. However, there is no evidence demonstrating that PeVYV could be transmitted by whiteflies. These data in combination with previous studies demonstrated that the tetra-interaction of virus–vector and non-vector–plants is common and probably dominates in natural and field ecosystems.

The volatiles emitted by virus-infected plants play a major role in determining the settling and feeding preference of vectors (Casteel et al., 2014; Mauck et al., 2012, 2018; Pan et al., 2021) and non-vector insects (Li et al., 2014; Wu et al., 2019). To fulfill immediate needs, viruses hijack plant volatiles to attract their vectors and enhance their spread (Shi et al., 2021). PeVYV is a phloem-restrictive virus (Filardo et al., 2024); however, it is well documented that poleroviruses could invade mesophyll tissues and be transmitted mechanically between plants during co-infection with umbravirus or potyvirus (Savenkov and Valkonen, 2001; Ryabov et al., 2001). Additionally, several poleroviruses, including beet western yellows virus (BWYV) and potato leafroll virus (PLRV), could be transmitted by mechanical inoculation (Hoffmann et al., 2001). These studies suggest poleroviruses likely infect the whole plant rather than remaining confined to phloem tissues in field conditions. Therefore, the PeVYV *P4* gene was constitutively expressed using the CaMV 35S promoter, and data in this study indicate that the P4 protein, encoded by PeVYV, likely reprogrammed *N. benthamiana* plant volatile pathways and co-attracted vector and non-vector insects (**Figure 1**). The hijacking of host volatiles to affect the insect’s preference for plants should be evolutionarily conserved in virus–vector and non-vector–plant interactions. The impact of changes in different viruses on volatile blends varies with different host plants; while some studies show that the blend of volatiles determines insect and their predator preferences for plants (Hu et al., 2020; Liu et al., 2021a; Munawar et al., 2023), virus-induced attraction may be attributable to a small number of, or potentially single, VOC including terpenoids, phenylpropanoid/benzenoid, fatty acid derivatives, branched-chain amino acid derivatives, and other special substrates (Ponzio et al., 2013; Zhou and Jander, 2022). The PeVYV P4 protein altered the biosynthesis of four novel VOCs (ketones, esters, and terpenoids), and these compounds demonstrated synergistic attraction to both aphids and whiteflies in the Y-tube olfactometer choice assays (**Figure 4**). This study and previous research demonstrated plant volatiles to be a hub in virus–vector and non-vector–plant interactions that could be used to develop novel strategies to manage viruses and pest-induced damage.

Phytohormones are central cellular signaling molecules with key functions in regulating plant immunity against biotic stimuli, including viruses, microbial pathogens, and insect herbivores (Pieterse et al., 2012). Virus, vector, and/or non-vector-infected plants activate different types of phytohormone-mediated responses (Pan et al., 2021). Previous research verified that vectors activate host phytohormone defense responses, which are adverse to viral replication/movement, such as a non-vector cotton bollworm infestation eliciting JA biosynthesis, benefiting begomovirus replication (Li et al., 2017). Dissimilar to a previous study, these data demonstrated that the virus–vector aphids have no effects on viral replication in the early stage (10–15 days), whereas non-vector Q-whiteflies enhance viral replication in the early stage, which is pivotal in PeVYV primary infection in plants (**Figures 2**, **5**, **6**). Moreover, in the late stage of viral infection (20–25 days), to counter the challenge posed by the plant defense response induced by rapid viral replication, PeVYV likely induces tZ biosynthesis via its vector aphids to enhance viral replication. Data from this study show that PeVYV evolved an ingenious strategy of co-attracting non-vector Q-whiteflies and vector aphids by hijacking host volatiles and regulating phytohormone biosynthesis of tZ to fine-tune viral infection. It seems reasonable to postulate that non-vectors could have co-evolved with viruses, vectors, and plants and might have a role in developing alternate approaches to managing plant viruses and pest infestations in the field.

This study describes studies of an aphid-vectored phloem-limited virus, PeVYV, and its encoded movement protein P4 that changes plant volatile emissions to co-attract vector aphids and non-vector Q-whiteflies and induce tZ biosynthesis to enhance viral infection. Herein, a hitherto previously uncharacterized natural phenomenon involving tetra-interaction among virus–vector/non-vector–host interactions is documented; the plant volatiles were subverted by the virus to deter vector and non-vector behaviors and mediate phytohormone biosynthesis to fine-tune viral infection. These data will pave the way to understanding the ecological insights to predict vector-transmitted viral infections and provide an alternative method of biological control for vector-borne diseases.

## Data Availability

The RNA-seq data have been deposited in NCBI, PRJNA749136 (https://www.ncbi.nlm.nih.gov/bioproject/?term=PRJNA749136). The LC-MS data of metabolome have been deposited in the OMIX, China National Center for Bioinformation/Beijing Institute of Genomics, Chinese Academy of Sciences (https://ngdc.cncb.ac.cn/omix: accession no. OMIX004523), BioProject accession number PRJCA018369 (https://ngdc.cncb.ac.cn/bioproject/browse/PRJCA018369PRJCA018369). The LC-MS data of phytohormone have been deposited in the OMIX, China National Center for Bioinformation/Beijing Institute of Genomics, Chinese Academy of Sciences (https://ngdc.cncb.ac.cn/omix: accession no. OMIX010046), BioProject accession number PRJCA039653, https://ngdc.cncb.ac.cn/bioproject/browse/PRJCA019763.
