# Supplementary material for: A viral movement protein mediates host volatile biosynthesis to co-attract vectors and non-vectors and enhances viral infection
Source: Front Plant Sci. 2025 May 16;16:1551362. doi: 10.3389/fpls.2025.1551362 (PMC12122504; doi:10.3389/fpls.2025.1551362)
Supplement: Supplementary file 1 [file DataSheet1.zip › Data Sheet 2 (1)/SI Appendix-revised1.docx]

**SI Appendix**

**Fig S1. Morphology of *Nicotiana tabacum* plants with constitutive expressing P4 gene.** (**A**) Detection of P4 gene inserted into plant genome by PCR using extracting total DNA from T2 generation of P4-OE *N. tobacco* plants. (**B**) The expression of P4 gene in T2 generation tobacco plants was detected by RT-PCR. M: DL2000 DNA ladder; WT: wild type of *N.tabacum* plants; CK: negative control using ddH_2_O as template. (**C**) Morphology of T2 generation of P4-OE *N tabacum* plants and wild type plants at 30 d. (**D**) Plant height. (**E**) Stem diameter. (**F**) Leaf length. (**G**) Leaf width. (**H**) SPAD. (**I**) A. (**D** to **I**) Bars represent means ± SD (n = 5) (ns, Student’s *t* test)

**Fig S2. Growth status and RT-PCR detection of chili peppers prefed by non-venomous aphids and venomous aphids.** (**A**) Morphology of chili peppers prefed by non-venomous aphids and venomous aphids, WT: healthy chilli pepper plants; A: nonviruliferous aphids prefed chilli pepper plants; A+V: viruliferous aphids of PeVYV prefed chilli pepper plants. (**B**) Specific detection of PeVYV by RT-PCR, M: DL2000 DNA ladder; PeVYV: chilli pepper infected by PeVYV detected by RT-PCR, P: positive control using total RNA of PeVYV infected plant as template; N: negative control using ddH_2_O as template.

**Fig S3.** **Effects of different treatments on plant growth and development.** (**A**) The morphology of plants affected by aphids and/or Q-whiteflies. (**B**) The plant height, leaf length, leaf width and stem diameter of plants affected by aphids and/or Q-whiteflies. (**C**) Plant height of PeVYV infection plants effected by aphids and/or whiteflys. (**D**) Leaf length of PeVYV infection plants effected by aphids and/or whiteflys. (**E**) Leaf width of PeVYV infection plants effected by aphids and/or whiteflys. (**F**) Stem diameter of PeVYV infection plants effected by aphids and/or whiteflys. Bars represent means ± SD (n = 5) (**ρ* < 0.05, ***ρ* < 0.01 and *** *ρ* < 0.005, Student’s *t* test).

**Fig S4. The phenylpropanoid biosynthesis pathway (nta00940) downloaded from KEGG (Kyoto Encyclopedia of Genes and Genomes, https://www.kegg.jp/pathway/map00940)**

**Fig S5. Phytohormones accumulation effected by aphids and/or whiteflies pre-feeded plants.** (**A**) Abscisic acid (ABA). (**B**) Indoleacetic acid (IAA). (A and B). Bars represent means ± SD (n = 5) (**ρ* < 0.05, ***ρ* < 0.01 and *** *ρ* < 0.005, Student’s *t* test).

**Fig S6. The mRNA levels of key genes involving phytohormones.** The key genes of *NtLOX* (**A**)、*NtAOS* (**B**)、*NtAOC* (**C**) involving in jasmonic acid (JA) biosynthesis; The key genes of *ICS1-det* (**D**)、*NtSGT* (**E**)、*PAL3* (**F**) involving salicylic acid (SA) biosynthesis; The key genes of *ACO1-det* (**G**)、*ACS1-det* (**H**)、*Ein2-det* (**I**) involving in 1-aminocyclopropane-1-carboxylic acid (ACC) biosynthesis; The key genes of *CYP735A1* (**J**), *CYP735A2* (**K**), *ABCG21* (**L**) involving in trans-zeatin (Tz) biosynthesis. Bars represent means ± SD (n = 5) (**ρ* < 0.05, ***ρ* < 0.01 and ****ρ* < 0.005, Student’s *t* test).

**Table S1 LC-MS/MS analysis of VOCs identified in wild type and P4-OE plants.**

**Table S2 Differentially biosynthesizing and releasing of VOCs induced in P4-OE plants.**

**Table S3 The differentially expressed genes (FDR < 0.05 and >= 2-fold change) involving in phenylpropanoid biosynthesis pathway.**

**Table S4 Primers used in this study**

**Fig. S1**

**
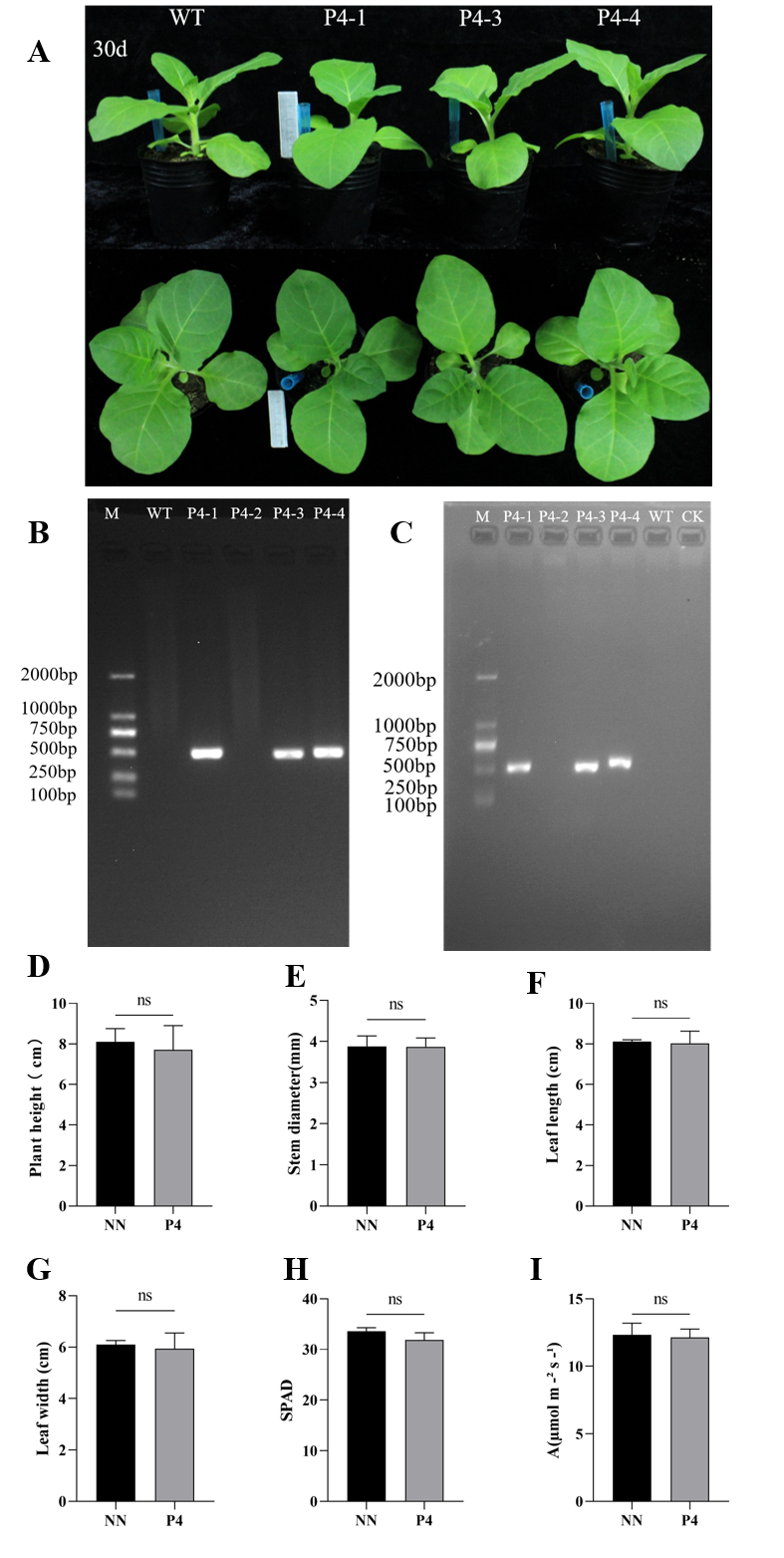
**

**Fig. S2**

**
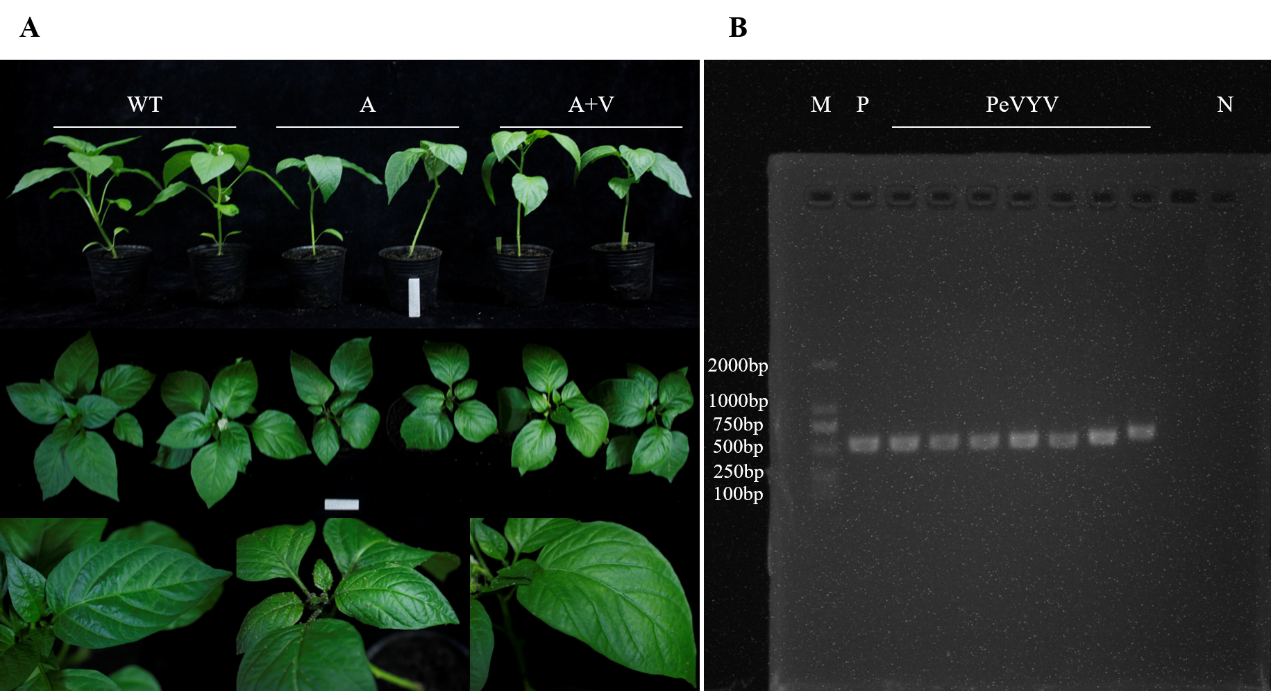
**

**Fig. S3**

**
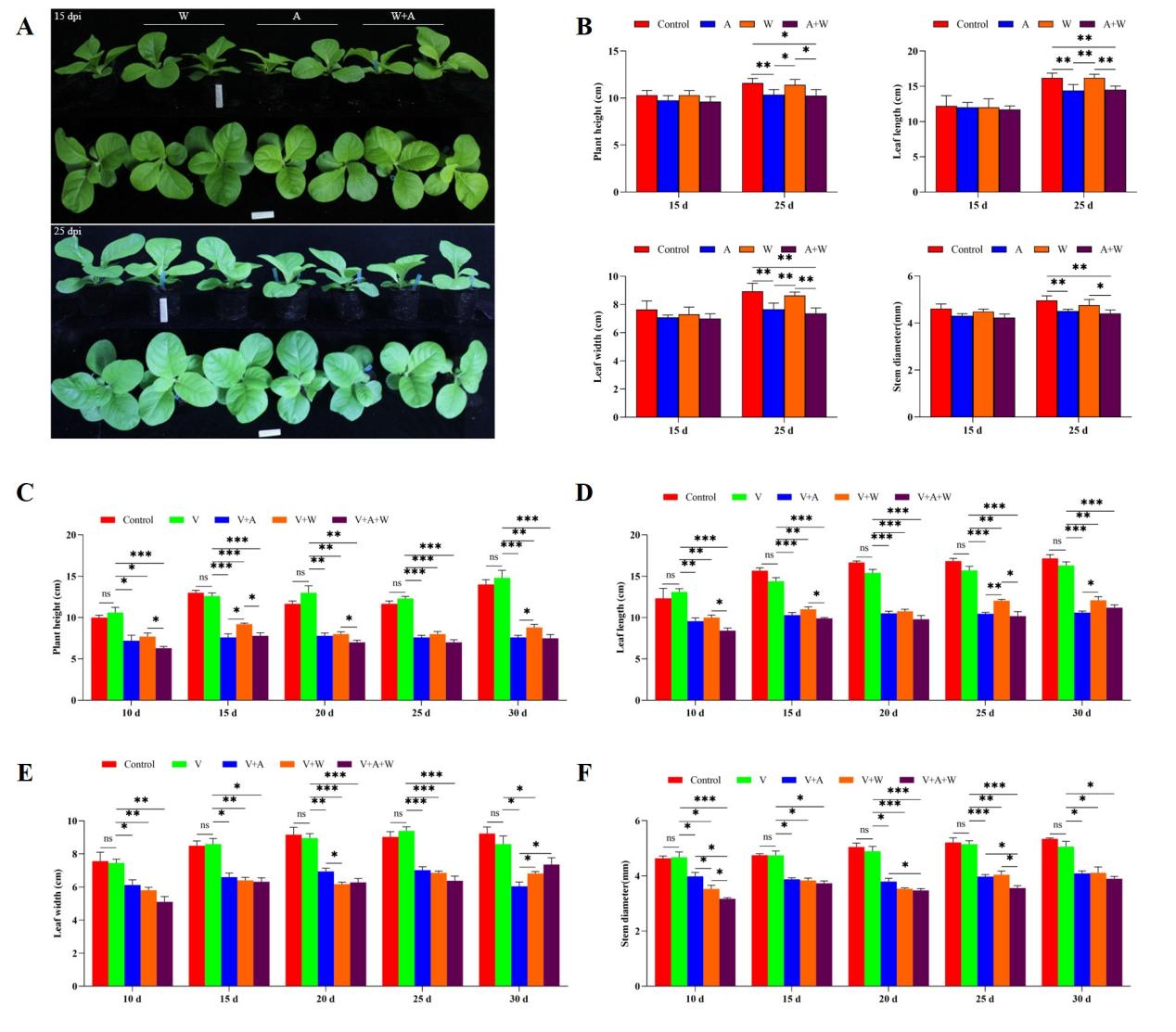
Fig. S4**


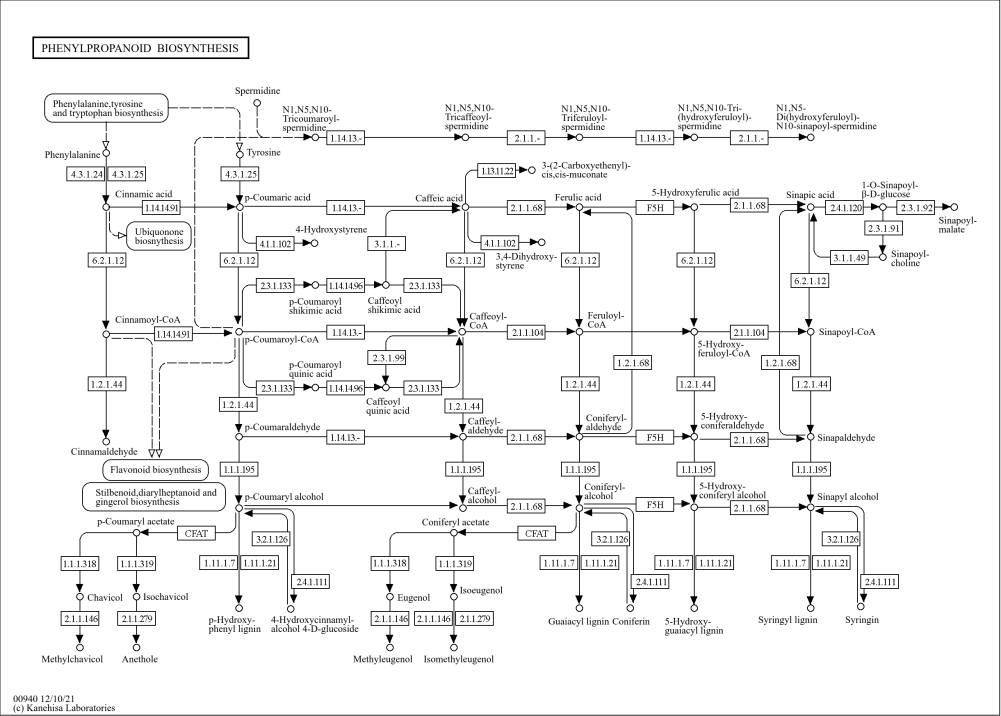


**Fig. S5**

**
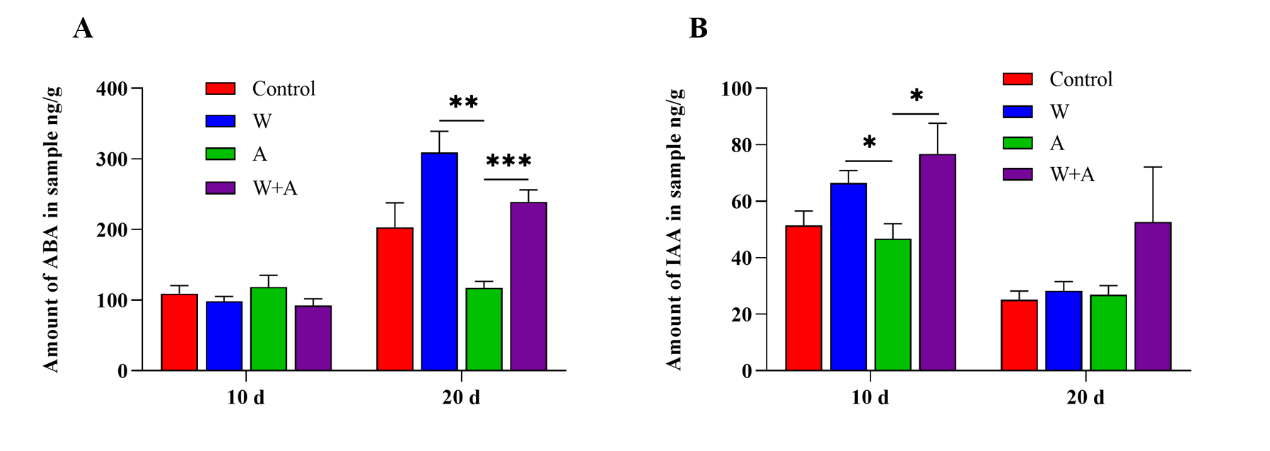
**

**Fig. S6**

**
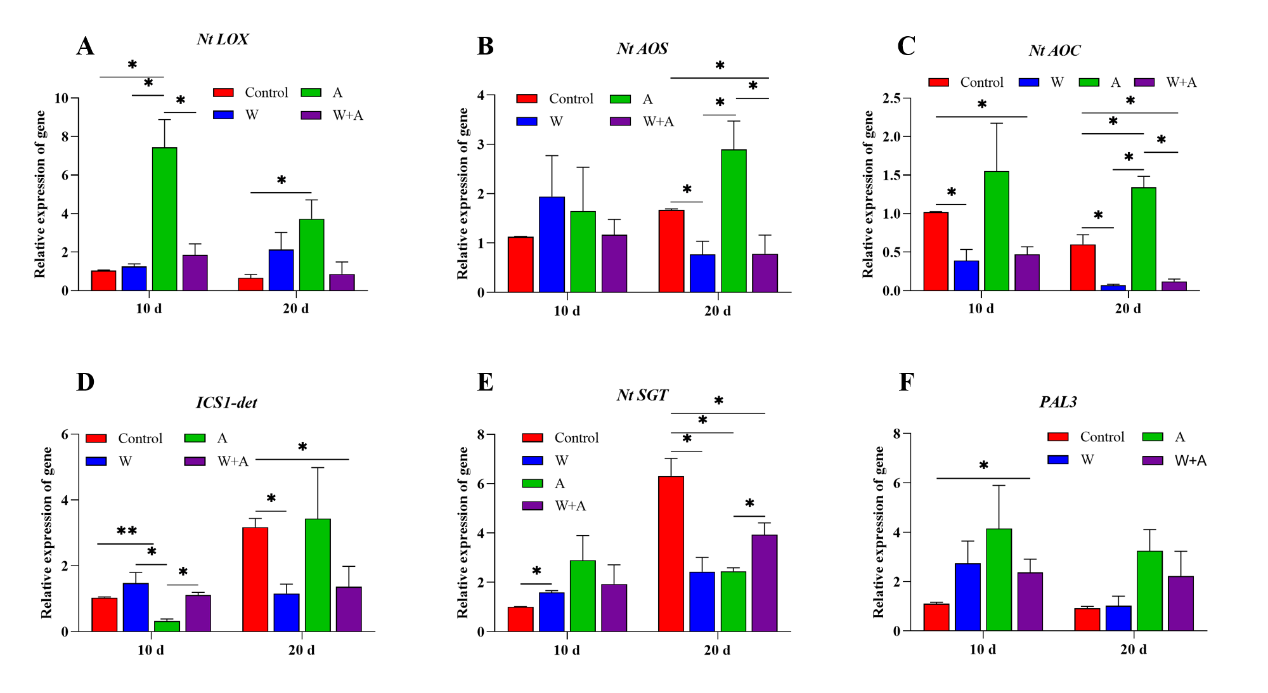

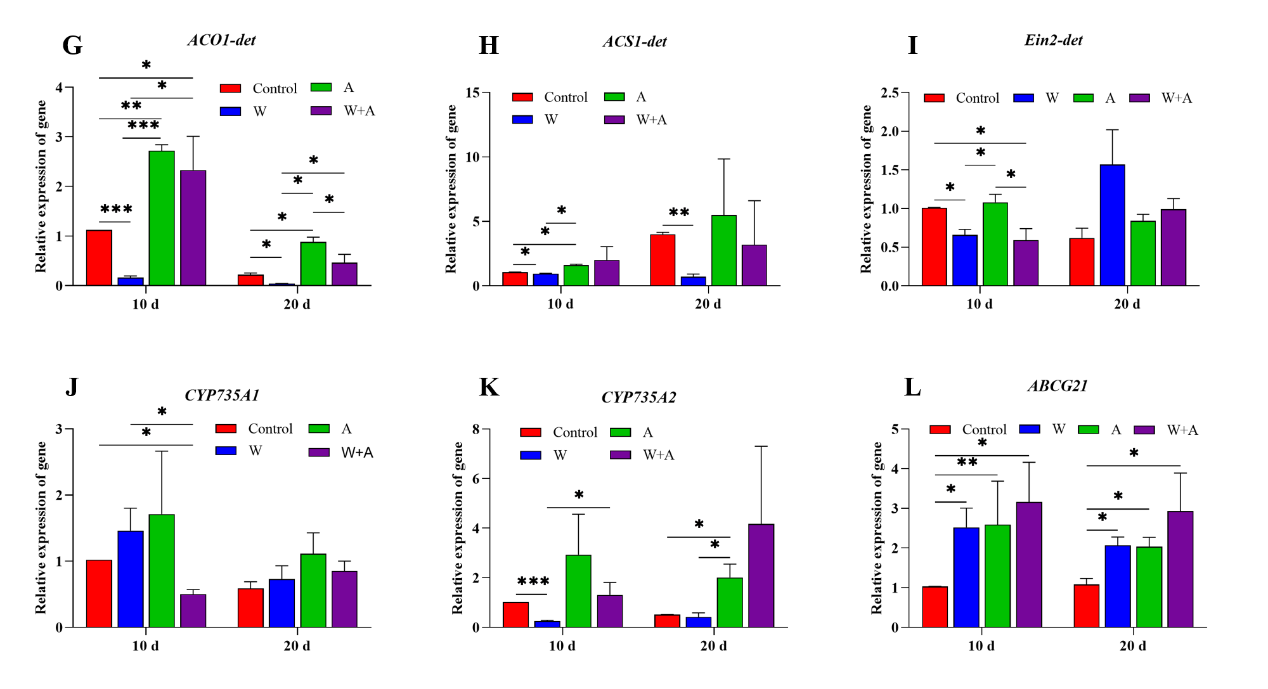
**
